# Predictors of mortality among patients with type 2 diabetes in Jordan

**DOI:** 10.1186/s12902-021-00866-8

**Published:** 2021-10-12

**Authors:** Fadia Abdallah Mayyas, Khalid Shaker Ibrahim

**Affiliations:** 1 Department of Clinical Pharmacy, Faculty of Pharmacy, Jordan University of Science and Technology, 3030, Irbid, 22110 Jordan; 2 Princess Muna Heart Institute, King Abdullah University Hospital, Division of Cardiac Surgery, Department of General Surgery, Faculty of Medicine, Jordan University of Science and Technology, Irbid, Jordan

**Keywords:** Diabetes mellitus, Mortality, Cardiovascular diseases, Jordan, Chronic kidney disease

## Abstract

**Background:**

Diabetes Mellitus (DM) is a common metabolic disease associated with increased risk of mortality.

**Objective:**

The aim of this study was to examine predictors of mortality among patients with type 2 diabetes in the north of Jordan.

**Methods:**

Electronic data files for diabetes patients admitted between the period of 2014–2018 at a tertiary center in the north of Jordan were reviewed. Patient’s characteristics, clinical and laboratory data, use of medications and mortality rate were collected.

**Results:**

Mean age of patients (*n* = 957) was 60.99 ± 0.37 (mean ± sem). Most of patients had multiple risk factors and underlying cardiovascular diseases (CVDs). Mortality rate was 10.1%. Univariate predictors of mortality included age, chronic kidney disease (CKD), acute kidney injury, hypertension, heart failure (HF), coronary artery disease, venous thromboembolism (VTE), stroke, atrial fibrillation (AF), and chronic obstructive pulmonary disease (COPD). As the number of CVDs increases, mortality rate also increases (Odd ratio 2.0, *p* < 0.0001). Use of insulin, aspirin, ACEi/ARBS, beta blockers, and diuretics were also associated with mortality. Fasting glucose and percentage of glycated hemoglobin were not associated with mortality. By multivariable logistic regression analysis adjusting for confounders and collinearity; age, HF, AF, COPD, VTE, and CKD were associated with mortality.

**Conclusion:**

Key risk factors of mortality are CVDs and CKD indicating that the primary step of management should focus on optimizing risk factors to prevent diabetes complications and death.

## Background

Diabetes Mellitus (DM) is a common metabolic disease associated with substantial increase of mortality [1]. DM is a significant risk factor for cardiovascular diseases (CVDs), kidney diseases and other complications [2]. In a national study in USA, DM was associated with a 16% increase in all-cause mortality and about 18% of CVDs mortality [2]. Myocardial infarction is the leading cause of mortality among diabetes patients [3]. Thus, measures to reduce death rely primarily on improving glycemic control to decrease DM complications and modifying risk factors especially in those with CVDs [3]. Early studies between 1990 and 2010 in the Middle East and North Africa found that both DM and CVDs were the leading causes of mortality; responsible for nearly 1 million deaths annually [4, 5]. In these countries, suboptimal diet and high blood pressure were the leading risk factors for cardiometabolic disease mortality; accounting for about 50–70% of deaths, followed by elevated body mass index (BMI) and fasting plasma glucose [6]. Recent data on risk factors of mortality among patients with diabetes are lacking in the Middle East region.

Jordan has one of the highest smoking and obesity prevalence rates in the region [7]. Both factors closely linked to risk of cardiometabolic diseases. DM is very common in Jordanian population accounting for signifcant mortality [8]. A previous study in Jordan has found that prevalence of type 2 DM and impaired fasting glucose was 17.1 and 7.8%, respectively, with about 54% of patients with unsatisfactory glycemic control [9]. No previous studies have assessd risk factors of mortality among patients with diabetes in Jordan.

The aim of this study was to examine risk factors and predictors of mortality among patients with type 2 diabetes at a primary tertiary center in the north of Jordan.

## Methods

Study participants.

This study includes patients with type 2 DM admitted to the outpatients’ internal medicine clinic at King Abdullah University Hospital (KAUH). Diabetes was established by fasting plasma glucose (FPG) ≥ 126 mg/dl (≥7 mmol/L) and/or glycated hemoglobin (HbA1c) ≥ 6.5% (≥48 mmole/mol) [10].

Patient’s medical files were reviewed electronically. All patients admitted to the hospital between 2014 and 2018 period with documented type 2 diabetes were included. Data were extracted retrospectively for each patient, including patient’s demographic, clinical, and laboratory characteristics. This study was approved by the institutional review board of KAUH and Jordan University of Science and Technology (JUST) (# 295/2014). All procedures were performed in accordance with the ethical standards of the Helsinki Declaration.

Patients underlying comorbidities were established according to standard guidelines and documented in patients’ electronic records. Hypertension (HT) was documented by a blood pressure of 140/90 or current use of antihypertensive medications [11]. Coronary artery disease (CAD) was documented as ≥50% stenosis in one of the main coronaries or positive stress testing [12]. Heart failure (HF) was established according to the ACC/AHA guidelines [13]. History of atrial fibrillation (AF) was established according to the ACC/AHA guidelines [14]. Venous thromboembolism (VTE) was established by laboratory tests and diagnostic imaging [15] as documented in the patients’ files. Chronic obstructive pulmonary disease (COPD) was established according to the Global Initiative for Chronic Obstructive Lung Disease (GOLD) [16]. Chronic kidney disease (CKD) was established by a decline in the glomerular filtration rate as described in the KDIGO 2012 clinical practice guidelines for the evaluation and management of CKD [17]. Moderate and severe albuminuria were documented regardless of presence of symptomatic CKD. Moderate increase in albuminuria (microalbuminuria) was established when at least two albuminuria readings were > 30 mg/dl in random urine samples that were 3 to 6 months apart, whereas severe albuminuria (macroalbuminuria) was documented when readings were > 300 mg/dl [18]. Laboratory data of lipids, glucose, kidney function and echocardiography were obtained and evaluated as risk factors of mortality. Last measured laboratory readings were considered for analysis.

Patients drug profile was revised to assess use of medications as predictors of mortality. All-cause mortality was documented electronically for diabetes patients who died in the period of 2014 to 2018.

### Statistical analysis

Data are presented as meam±sem for continuous variables and percentages for categorical variables (*n* = 957). Univariate analysis for predictors of mortality was carried out using chi-square test for dichotomous variables, whereas logistic regression was used for categorical variables with more than 2 levels and continuous variables. Multivariable logistic regression using stepwise selection was used to assess independent predictors of mortality among diabetes patients. Univariate predictors of *p* value < 0.25 were entered in the model with an exit *p* value of > 0.2. Collinearity was tested by evaluating the correlation between independent variables. Statistical analysis was performed using JMP11, USA. A *p* value of < 0.05 was considered statistically significant.

## Results

### Patients characteristics

Table 1 represents characteristics of study population. A total of 957 patients with type 2 DM were included in this study during the period of 2014 to 2018. Average age of patients was 60.99 ± 0.37 (mean ± sem). About half of them were male and most of them had multiple risk factors and underlying morbidities. HT, CAD, peripheral artery disease (PAD), HF, stroke, history of AF, and VTE were present in 82.4, 49.1, 3.9, 10.4, 12.4, 4.9 and 2.2% of patients; respectively. Body mass index was documented for only 171 patients with mean of 32.33 ± 1.49 (Kg/m2).

**Table 1:** Patients’ characteristics

	***N*** = 957
**Patients characteristics**	
Age, years	60.99 ± 0.37
Male gender	482 (50.40)
BMI (Kg/m2), *n* = 171	32.33 ± 1.49
Hx of hypertension	789 (82.44)
Hx of heart failure	99 (10.35)
Hx of CAD	470 (49.11)
Hx of PAD	38 (3.97)
Hx of stroke/TIA	119 (12.43)
Hx of VTE	21 (2.19)
Hx of AF	47 (4.91)
Hx of CKD	115 (12.02)
Hx of AKI	163 (19.47)
Dialysis	24 (2.50)
Hx of COPD	29 (3.03)
**Diabetes mellitus**	
Type 2	957 (100.0)
Diabetic nephropathy	41 (4.28)
DKA	12 (1.43)
Controlled DM, HbA1c < 7%	63 (6.65)
Duration of DM, yrs.	7.93 ± 0.21
NA	42 (4.38)
**Hx of smoking**	
Current smoking	149 (15.56)
X smoker	76 (7.94)
NA	424 (44.30)
**Mortality**	97 (10.14)
**Lab tests (mg/dl)**	
Low Density Lipoprotein (LDL)	134.82 ± 2.17
High Density Lipoprotein (HDL)	42.09 ± 0.64
Triglyceride	267.77 ± 8.29
Total cholesterol (Tch)	207.04 ± 2.52
Fasting plasma glucose	265.58 ± 3.71
HbA1c % (mmole/mole)	10.19 ± 0.078
Creatinine level (mg/dl)	1.59 ± 0.060
Microalbuminuria	304 (31.77)
Macroalbuminuria	154 (16.11)
**Echocardiographic data**	
Left ventricular ejection fraction (LVEF), %	55.13 ± 0.003
atrial size, cm	3.88 ± 0.0175
**Use of medications, n (%)**	
ACEIs/ARBs	741(77.43)
Aldosterone antagonists	80 (8.35)
Thiazide diuretics	350 (36.61)
Loop diuretics	372 (38.87)
Beta blockers	693 (72.41)
Digoxin	67 (7.00)
Statins	818 (85.47)
Oral Hypoglycemic drugs	926 (96.76)
Insulin	604 (63.11)
Aspirin	793 (82.86)

Values are presented as mean ± SEM, and percentages N (%) for categorical variables. *BMI* body mass index, *CAD* coronary artery disease, *PAD* peripheral artery disease, *TIA* transient ischemic attack, *AF* atrial fibrillation, *VTE* venous thromboembolism, *CKD* chronic kidney disease, *AKI* acute kidney injury, *COPD* chronic obstructive pulmonary disease, *Hx* history, *HbA1c* glycosylated hemoglobin, *DKA* diabetic ketoacidosis, *ACEI* angiotensin converting enzyme inhibitor, *ARBs* angiotensin-II receptor blockers, *NA* not available. Unit for lipids and glucose is mg/dl

Average left ventricular ejection fraction (LVEF) was normal among patients (mean ± sem = 55.13 ± 0.003). About 3.03% of patients had COPD. One third of patients had microalbuminuria (31.8%), with 12% had CKD and 19.5% had acute kidney injury (AKI). All patients were on oral hypoglycemic drugs (OHA, 96.8%) and/or insulin therapy (63.1%). Most of patients were under-controlled and 93.4% of them had an HbA1C ≥7% (≥53 mol/mole). All patients were on multiple medications to control DM or underlying comorbidities. Aspirin, statin, angiotensin converting enzyme inhibitors/ receptor blockers (ACEi/ARBs) and beta blockers were used by about 82.9, 85.5, 77.4 and 72.4% of patients; respectively. A total of 97 patients died between 2014 and 2018 with a mortality rate of 10.1%.

### Predictors of mortality among diabetes patients

We have evaluated factors associated with increased mortality (Table 2). By univariate analysis, age (Odd ratio (OR) 1.06), HT (OR 7.54), HF (OR 3.95), CAD (OR 1.62), VTE (OR 3.71), stroke (OR 2.47), AF (OR 5.28), and COPD (OR 3.59) were all associated with increased mortality rate (*p* < 0.05). As the number of cardiovascular comorbidities increases, mortality rate also increases (OR 2.00, *p* < 0.0001). Current smoking, gender and PAD were not associated with mortality (*p* > 0.05). Diabetic nephropathy is a common DM complication and was associated with mortality, similar to macro and microalbuminuria (*P* < 0.0001). Both CKD (OR 4.61) and AKI (OR 8.75) were strongly associated with mortality including those who underwent dialysis (*p* < 0.0001). Diabetes ketoacidosis (DKA) is an acute complication of DM and was also associated with mortality. Neither lipid profile nor glucose or HbA1c% levels were associated with mortality (*p* > 0.05). Because 94% of patients were under controlled with mean HbA1c% of 10.19 ± 0.078, we have stratified patients based on HbA1c% values into categories and evaluated their relation to mortality. HbA1c% ranges were: < 8, 8- < 9.5, 9.5- < 11, 11- < 13, ≥13. No association was found between any of the HbA1c% categories and mortality, *p* < 0.05 for all. Duration of DM in years was not associated with mortality (*p* = 0.24). We have further explored relation of duration of DM to mortality by stratifying patients into categories ≤6 vs. > 6 years. DM duration > 6 years was associated with mortality (*p* = 0.0183, OR 1.67 (95% CI: 1.09–2.57). Patients with significant increase in glucose level are usually controlled by insulin therapy. Use of insulin (OR 1.77, *p* = 0.0167) and use of combination of insulin and OHA therapy (OR 1.68, *p* = 0.0252) were associated with mortality, whereas use of OHA alone is associated with reduced mortality (OR 0.583, *p* = 0.0248). Reduced LVEF and enlarged left atria were both associated with mortality (*p* < 0.0001). Patients with DM have several underlying CVDs that require management by drug therapies. Use of beta blocker, aldosterone antagonists, digoxin, loop diuretics and aspirin were all associated with mortality (*p* < 0.05), whereas use of statin and ACEi/ARBs were not associated with mortality (Table 2).

**Table 2:** Univariate predictors of mortality among patients with type 2 diabetes

Response = Mortality, ***N*** = 957			
	Confidence interval (95%)	***P*** value	Odd Ratio
Age, yrs.	1.0389–1.0831	< 0.0001*	1.0630
Male Gender	0.6908–1.6004	0.8147	1.0515
BMI (Kg/m2), n = 171	0.9547–1.0621	0.7221	1.0096
Current smoking	0.4492–1.4399	0.4630	0.8043
Hypertension	4.3030–10.754	0.0007*	7.5423
HF	2.3724–6.5696	< 0.0001*	3.9479
CAD	1.0547–2.4793	0.0264*	1.6171
PAD	0.6947–4.1895	0.2386	1.7060
VTE	1.4064–9.8091	0.0046*	3.7142
Stroke/TIA	1.4797–4.1347	0.0004*	2.4735
AF	2.7713–10.0684	< 0.0001*	5.2823
COPD	1.5455–8.3445	0.0016*	3.5912
CKD	2.8518–7.4478	< 0.0001*	4.6087
Number of CVD comorbidities	1.6666–2.4219	< 0.0001*	2.0005
Diabetic nephropathy	2.2950–9.2144	< 0.0001*	4.5986
Macroalbuminuria	2.9248–7.1905	< 0.0001*	4.5860
Microalbuminuria	1.7395–4.0673	< 0.0001*	2.6599
AKI	5.5478–13.8176	< 0.0001*	8.7554
Dialysis	1.9739–11.3898	0.0001*	4.7415
DKA	0.0901–5.5301	0.7390	0.7095
DM duration	0.9868–1.0496	0.2434	1.0189
DM duration, ≤ > 6 yrs.	1.0940–2.5705	0.0183*	1.6696
Controlled DM	0.4938–2.5243	0.7910	1.1165
LVEF	0.0014–0.1248	0.0001*	0.0129
LA size, cm	2.6298–6.3121	< 0.0001*	4.0400
Cr level	1.2072–1.4613	< 0.0001*	1.3246
LDL	0.9970–1.0038	0.7178	1.0006
HDL	0.9756–1.0053	0.3166	0.9921
Triglyceride	0.9979–1.0002	0.1734	0.9991
Total Cholesterol	0.9971–1.0030	0.8960	1.0001
Fasting plasma glucose	0.9967–1.0011	0.4035	0.9990
HbA1c, %	0.9714–1.1523	0.1865	1.0591
Use of Insulin	1.1038–2.8562	0.0167*	1.7756
Insulin alone	0.3733–4.3551	0.6975	1.2750
Use of OHA	0.3145–3.5348	0.9315	1.0544
OHA alone	0.3623–0.9379	0.0248*	0.5830
Combination of insulin and OHA	1.0627–2.6654	0.0252*	1.6830
ACEi/ARB	0.8771–2.6831	0.1311	1.5340
Beta blockers	1.5761–5.4684	0.0004*	2.9358
Aldosterone antagonists	1.3569–4.4480	0.0023*	2.4567
Thiazide Diuretics	0.9824–2.2912	0.0591	1.5003
Loop Diuretics	2.5343–6.3904	< 0.0001*	4.0639
Statins	0.7100–2.6321	0.3478	1.3671
Digoxin	1.5325–5.2717	0.0006*	2.8424
Ion channel blockers	0.8954–8.7702	0.0649	2.8023
Aspirin	1.0594–4.3620	0.0303*	2.1497

Odd ratio is per unit change in regressor. * is a *p *value <0.05. *BMI* body mass index, *CAD* coronary artery disease, *PAD* peripheral artery disease, *TIA* transient ischemic attack, *AF* atrial fibrillation, *VTE* venous thromboembolism, *CKD* chronic kidney disease, *AKI* acute kidney injury, *COPD* chronic obstructive pulmonary disease; number of cardiovascular morbidities include HT, HF, CAD, PAD and stroke, *Hx* history, *HbA1c* glycosylated hemoglobin, *DKA* diabetic ketoacidosis, *ACEI* angiotensin converting enzyme inhibitor, *ARBs* angiotensin-II receptor blockers

### Independent predictors of mortality among diabetes patients

We have performed multivariable logistic regression analysis to evaluate independent risk factors associated with mortality adjusting for possible confounders (Table 3). By step wise analysis, both LVEF and LA size were associated with mortality (< 0.05) but were not included in the model due to collinearity with HF. As creatinine level, albuminuria, nephropathy and dialysis were collinear with CKD, they were also excluded from the model. Both use of aldosterone antagonists and loop diuretics were associated with mortality, but due to collinearity with HF and HT, they were not included in the final model. By multivariable logistic regression adjusting for other variables and collinearity, age, HF, AF, COPD, VTE, and CKD were significantly and independently associated with mortality. History of AKI was strongly and independently associated with mortality (OR 5.82, *p* < 0.0001). However, because presence of CKD, HT, HF or stroke could predispose to AKI, AKI was removed from the model to avoid overlap between them. History of stroke/TIA was marginally significant. Use of insulin and use of combination of insulin+ OHA were associated with mortality, however, insulin use was entered in the final model to avoid collinearity. HT and insulin use were not associated with mortality in the final model (Table 3). Adjusting for other variables, use of beta blocker and duration of DM > 6 years were not associated with mortality and were removed from the model (*p* = 0.4183, *p* = 0.2799; respectively). In another model, increased number of CVD indications was found significantly associated with mortality (*p* < 0.0001, OR 1.7619, 95% CI: 1.457–2.1616).

**Table 3:** Multivariate predictors of mortality among patients with type 2 diabetes

Response = Mortality, ***N*** = 957			
	Confidence interval (95%)	***P*** value	Odd Ratio
Age, yrs.	1.0125–1.0610	0.0028*	1.0362
Hypertension	0.9158–6.4258	0.1106	2.1747
Heart failure	1.1585–3.7228	0.0121*	2.1065
Venous Thromboembolism	0.9932–8.7748	0.0372*	3.1326
Stroke/TIA	0.9213–2.9071	0.0818	1.6624
Atrial Fibrillation	1.1680–5.3020	0.0155*	2.5349
COPD	1.1298–7.4443	0.0199*	3.3044
Chronic Kidney Disease	1.6031–4.6813	0.0002*	2.7593
Use of Insulin	0.8860–2.5193	0.1433	1.4749

Odd ratio is per unit change in regressor. *TIA* transient ischemic attack, *COPD* Chronic obstructive pulmonary disease

## Discussion

In the present study, we have assessed a broad number of characteristics and clinical data that might be associated with increased mortality among patients with type 2 diabetes. The prevalence of DM and its associated death are rapidly increasing in Jordan [8, 9]. Our center is a tertiary center that provides health care for a significant number of DM patients in the Northern area of Jordan. Laboratory data of key parameters such as lipid and kidney function were assessed, in addition to use of medications.

All included patients have type 2 DM, which is associated with obesity that is common in Jordan [7]. However, obesity as indicated by BMI was not associated with mortality in our study probably because it is documented for small number of patients. DM promotes atherosclerosis and HT predisposing into HF, arrhythmia and thrombosis. Diabetes mellitus remains significantly associated with all-cause of CVD mortality [19]. CVDs are associated with 3- to 4-fold higher risk of mortality in patients with DM relative to those without DM [19]. Our findings suggest that CVDs including HT, HF, stroke, VTE and AF diseases are the primary predictors of mortality among patients, and the odds of mortality increase with increased number of underlying CVDs. Consistent with our findings, previous studies showed that CVDs account for most of mortality in DM patients [4, 5]. Thus, primary prevention of CVD is a major goal among patients with DM to reduce risk of death [20]. In USA, improvement in the control of CVDs risk factors and preventive practices between 1990 to 2010 [21] was associated with a decline in the rates of DM complications [22]. In a nationwide registry, in-hospital mortality between 1997 and 2010 period remarkably decreased in DM patients, from 19.9 to 9.0%, with a decrease of 6% per year of admission [2]. Although increased awareness of DM diagnosis and management is expected to improve DM outcomes and mortality rates, mortality is still significant.

Therapeutic management of DM is multifactorial process that involves targeting serum glucose, cholesterol, blood pressure goals, in addition to modifications of life style habits [23]. The percentage of HbA1c is an indicator of long term glycemic control. Patients with DM should maintain a percentage of < 7% HbA1c [24]. Increased gradient of mortality rate is associated with increased HbA1c% from > 6 to 6.9% [19]. Interestingly, it has been found that aggressive glycemic control may also increase mortality in elderly patients [25]. However, in our study, glycemic control as indicated by the increase in HbA1c% values or categories was not associated with mortality. It is important to note that 94% of our patients were under-controlled as documented by their last measured HbA1c%. The lack of association of HbA1c% with mortality may be due to the lack of wide distribution of patients over categories and limited number of controlled patients. Future studies with larger sample size may reveal a potential association.

Increased duration of diabetes is associated with development and progression of complications and comorbidities [26]. The increase in DM duration in years was not associated with mortality possibly because mean duration in our study was not long enough to promote complications. However, duration of more than 6 years was associated with mortality relative to duration less than 6 years by univariate analysis but not multivariate analysis. It has been found that longer diabetes duration > 5, 10 and 15 years is associated with proportional increase in odds of CVDs risk and mortality relative to patients with duration < 5 years [26].

Hyperglycemia promotes metabolic changes mediating microvascular diseases secondary to atherosclerosis and other complications including neuro and nephropathy [23]. These metabolic changes involve the production of advanced glycation end products, oxidative stress and histological changes that promote glomerular sclerosis. Nephropathy is one of the most prevalent chronic complications of DM [23]. Interestingly, microalbuminuria was found associated with increased arterial stiffness, left ventricular mass and atherosclerosis in patients with type 2 DM [27]. DM is one of the most common causes of end-stage renal disease [28]. Patients with DM and CKD are prone to hospitalization, and both AKI and CKD account for significant component of expenditure on medical care [29]. Our results documented that CKD and AKI are significant predictors of mortality in DM patients. Step wise analysis showed that microalbuminuria also predicts mortality, indicating the value of early diagnosis of nephropathy in DM patients to provide the optimal management to slow and prevent the development and the progression of CKD and AKI.

Previously, it was found that treatment with insulin with or without oral medications was a significant risk factor for all-cause and CVD mortality [30]. Similarly, by univariate analysis, we observed that use of insulin and combination of insulin with oral medications were associated with mortality, whereas use of oral medications alone is associated with reduced mortality. However, this relationship was not statistically significant when adjusting to potential confounding of other factors in the multivariable model. Although use of insulin provides effective glycemic control, it is usually used when glycemic control is not achieved by oral medications indicating disturbed glycemic status. Use of insulin may also increase risk of weight gain, hypoglycemia, metabolic syndrome and CVDs leading to mortality, especially in type 2 DM with insulin resistance [31]. Future studies with larger sample size should provide more understanding of this relationship.

Increased mortality rate is observed among patients using diuretics, β-blockers, and other antihypertensive medications. These medications are usually prescribed for patients with elevated and persistent HT and for patients with underlying CVDs, which are risk factors of mortality. By univariate analysis, many of used medications were associated with mortality including use of beta blockers, diuretics, and aldosterone antagonists. However, due to collinearity and confounding by comorbidities, they were not associated with mortality when included in the multivariate model adjusting for underlying diseases.

### Limitations of the study

This is a cross sectional study of patients with diabetes with retrospective data collection. Future follow up cohort studies are recommended to evaluate predictors of mortality over time. Our study evaluated patients’ comorbidities, lab data and use of medications. Evaluation of other factors such as family income and family history of CVDs is important to better understand predictors of mortality. Most of our patients were old, and age is associated with cardiac and metabolic diseases. Future studies should include patients with younger ages. The study is limited by the lack of a control group. Future controlled studies with larger sample size are recommended.

## Conclusion

In a large study of type 2 DM patients in the north of Jordan, we found that key risk factors of mortality are CVDs and kidney diseases indicating that primary step of management should focus on optimizing risk factors to prevent diabetes complications and death.

## Data Availability

Data are presented in the manuscript. Any additional data will be available upon request from the corresponding author.
